# Structure Elucidation of a Novel Polysaccharide Isolated from *Euonymus fortunei* and Establishing Its Antioxidant and Anticancer Properties

**DOI:** 10.1155/2024/8871600

**Published:** 2024-05-24

**Authors:** Yu Luo, Hongtao Chen, Chunxi Huang, Shujia He, Qilong Wen, Danzhao Cai

**Affiliations:** ^1^Guangxi Key Laboratory of Bio-Targeting Theranostics, Nanning 530021, China; ^2^Department of Biochemistry and Molecular Biology, Guangxi Medical University, Nanning 530021, China; ^3^Key Laboratory of Biological Molecular Medicine Research, Guangxi Medical University, Education Department of Guangxi Zhuang Autonomous Region, Nanning 530021, China; ^4^Guangxi University of Chinese Medicine Bainianle Pharmaceutical Co., Ltd, Nanning 530000, China; ^5^Department of Pharmacy, The First Affiliated Hospital of Guangxi Medical University, Nanning 530021, China

## Abstract

*Euonymusfortunei* polysaccharides (EFPs) have not been extensively investigated yet in terms of their extraction and biological activity. The orthogonal experimental design was employed in this study to evaluate the optimum yield of EFPs. A maximum yield of 2.63 ± 0.23% was attained using material-liquid ratios of 60 mL/g, extraction temperature of 80°C, ultrasonic power of 144 W, and extraction time of 75 mins. The polysaccharide content reached 53.47 ± 0.31% when deproteinized thrice. An analysis of monosaccharide composition revealed that these polysaccharides consist of Gal, Glc, Man, Fuc, and Rha with a molar ratio of 7.14 ∶ 23.99 ∶ 6.29 ∶ 6.55 ∶ 1.00, respectively, in EFPs. Subsequently, the *in vitro* scavenging capacities of 2,2-diphenylpicrylhydrazyl (DPPH) and ·OH and superoxide anion radicals, along with the reducing power of EFPs, were studied. Results revealed that EFPs have higher antioxidant activity, particularly ·OH scavenging, as well as reducing power, as compared to *Astragalus* polysaccharides (ASPs) and *Lycium barbarum* polysaccharides (LBPs). The Cell Counting Kit-8 (CCK-8) method was used to evaluate the effects of different concentrations of polysaccharides on SKOV3 cell proliferation, and the results revealed their inhibition at concentrations in the range of 200–800 *μ*g/mL. In addition, findings from flow cytometry further confirmed that EFPs blocked the cell cycle at G0/G1 and S phases and induced SKOV3 cell apoptosis. In a word, EFPs could be exploited and used further based on the experimental results from this study.

## 1. Introduction

*Euonymus fortunei* (*E. fortune*) has been frequently used in traditional Chinese medicine for maintaining health and treating diseases since ancient times. Modern medical research also shows that it contains various active ingredients such as polysaccharides, dulcitol, flavonoids, catechins, and triterpenoids [1–3]. Polysaccharides are among the main bioactive substances with various effects such as improving immunity, antitumor, antifatigue, liver protection, blood glucose lowering, antioxidation, and antiaging [4, 5]. However, reports on the study of polysaccharides from *E. fortunei* are very limited. It is necessary to clarify its pharmacological action, monosaccharide composition, and antioxidant effects and further study the relevant mechanisms.

In females, ovarian cancer is one of the three prevalent malignant tumors of the reproductive system, which has the highest mortality rate among gynecological malignant tumors [6]. Treatment strategies for ovarian cancer involve surgical resections and systemic pharmacological treatment. Cisplatin and paclitaxel are routinely used drugs, but their application has difficulties because the tumor patient would suffer a series of adverse reactions. Additionally, natural plant-based drugs play a crucial role in the chemotherapy of malignant tumors [7–12]. Hence, an exploration of low-toxicity and high-efficiency antitumor drugs of plant origin is gaining huge interest in tumor treatment. Currently, a growing number of researchers have realized that cell apoptosis induction, as well as affecting cell cycle, can be a new method to treat tumors, which could be achieved via the mechanism of inhibiting tumor cell proliferation and promoting apoptosis. Likewise, for ovarian cancer, Zhou et al. [13] have reported that a traditional Chinese medicine from *Salvia miltiorrhiza*, *tanshinone IA*, could considerably inhibit *in vitro* cell proliferation and *in vivo* tumor growth via apoptosis induction and autophagy promotion through inactivating the PI3K/AKT/mTOR pathway. Based on these findings, our current research focused on using ultrasonic extraction and alcohol-based precipitation for extracting crude polysaccharides from *E. fortunei*, where an extraction procedure has been optimized via orthogonal experiments, followed by column chromatography-based EFP purification. In the initial identification of *E. fortunei*, polysaccharide content was determined, and we also analyzed the monosaccharide composition of EFPs. The antioxidant activities of EFPs were evaluated, and antiovarian adenocarcinoma activity was studied *in vitro*. It is anticipated that this study will serve as a basis to evaluate the clinical application values of EFPs. Thus, suppose it could be proven that EFPs could treat ovarian cancer by regulating the body's immunity, cytotoxicity, and cell proliferation. In that case, it is expected to overcome the limitations of high side effects, easy drug resistance, and low survival rate of patients caused by chemotherapy drugs such as cisplatin or paclitaxel in the treatment of tumors.

## 2. Materials and Methods

### 2.1. Materials

**Figure 1:**
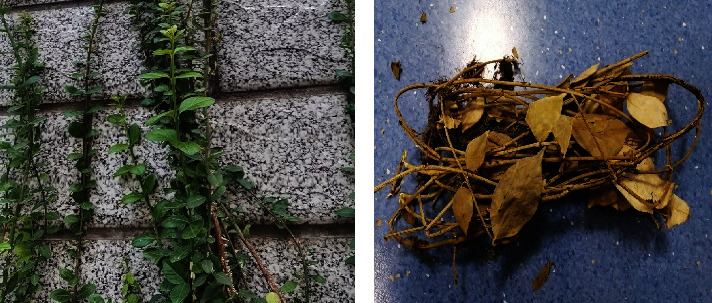
The plant of *E. fortunei*. (a) The fresh plant. (b) The dried plant.

*E. fortunei* was provided by the Pharmaceutical Factory of Guangxi University of Traditional Chinese Medicine (shown in Figure 1) and identified as genuine *E. fortunei* by Prof. Wei Songji from the Pharmaceutical Factory of Guangxi University of traditional Chinese medicine. The whole herb was crushed through an 80-mesh sieve using a grinder to obtain a fine powder, dried to a constant weight, and placed in a dryer till further use. The AB-8 macroporous adsorption resin, monosaccharide standards, and *Astragalus* and *Lycium barbarum* polysaccharides were all purchased from Beijing Solarbio Science & Technology Co., Ltd. (Beijing, China). Both the superoxide anion free radical detection kit and the hydroxyl radical detection kit were provided by Nanjing Jiancheng Biotechnology Co., Ltd. (Nanjing, China). The rest of the chemicals used for the tests were of analytical and chromatographic grade and were procured from regional chemical vendors in China. The SKOV3 cells were procured from the Chinese Academy of Sciences (Shanghai, China). The RPMI 1640 and fetal bovine serum were purchased from Gibco (Grand Island Biological Company, NY, USA). Other agents such as penicillin-streptomycin liquid and trypsin were brought from Solarbio Science & Technology Co., Ltd. (Beijing, China). The CCK-8 and fluorescein isothiocyanate-conjugated Annexin V (Annexin V-FITC)/propidium iodide (PI) apoptosis assay kits were procured from DOJINDO (Shanghai, China) and BD (Becton, Dickinson and Company, NJ, USA), respectively.

### 2.2. Sample Preparation of EFPs

#### 2.2.1. Single-Factor Experiment (SFE)

**Figure 2:**
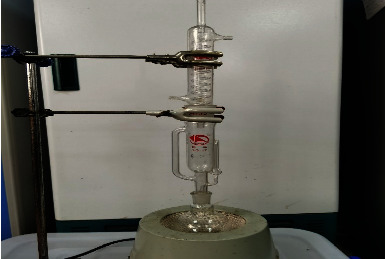
Soxhlet extractor device diagram.

Fifteen grams of finely dry powder was defatted with 300 mL of anhydrous ethanol at 95°C for 5 h using a Soxhlet extractor (shown in Figure 2) and then placed in the dryer until a constant weight was achieved. The constant weight powder was mixed with sterilized water before being extracted using the ultrasonic extractor. After filtration by vacuum pumping, the water extract was collected and then concentrated to 1/5th of the initial volume using a rotary evaporator at 55°C, followed by centrifugation at 5,000 rpm for 10 mins. The chloroform (1 : 5 diluted) was added to the collected supernatant, which was placed into the separating funnel to remove the protein. Absolute ethanol was slowly added while stirring to the collected supernatant, making the final ethanol concentration of the mixture reach 80%. Afterward, the solution was cooled at 4°C for 24 h, and then, centrifugation was carried out at 5,000 rpm for 10 mins for precipitate collection. Next, the residue was washed with ethanol and acetone. The crude polysaccharide sample was obtained by dryer drying (50°C) and then kept in a desiccator till further use. For further improving the yield of crude polysaccharide extraction from *E. fortunei*, the following five parameters were considered: material-liquid ratio, extraction temperature, ultrasonic power, and extraction times. The different values of these factors used for optimization involved the material-liquid ratios (20, 30, 40, 50, 60, and 70 mL/g); extraction temperatures (40, 50, 60, 70, 80, and 90°C); ultrasonic power (144, 198, 252, 306, and 360 W); and extraction time at 15, 30, 45, 60, and 90 mins. By using the measured EFP content, the extraction rate (ER) has been computed.

#### 2.2.2. Orthogonal Test Design for Optimization

Based on the outcomes of SFE, the following four parameters were used: extraction temperatures, ultrasonic power, material-liquid ratios, and extraction time, which were selected in an orthogonal test to optimize the optimal extraction process. Then, the orthogonal experiment was carried out following Table 1. All experiments were conducted in triplicates.

#### 2.2.3. EFP Purification

The crude polysaccharides were resuspended in the deionized water to a suitable volume and then injected into an AB-8 macroporous adsorption resin chromatography column (2.5 × 100 cm). Elution was carried out using a gradient of water and 5% and 15% ethanol solutions with a flow rate of 1 BV/h, whereas eluent collection was performed at 10 mL per tube with the aid of an automated collector. To determine the polysaccharide content in each tube, an elution curve was generated, using the phenol-sulfuric acid method. Fractions containing peak components were subjected to concentration, freezing, and drying.

### 2.3. Estimation of Polysaccharide Content

First, for the glucose standard curve, 0, 0.04, 0.06, 0.08, 0.1, 0.2, and 0.4 mL from glucose stock (1 mg/mL) were added to seven dry grinding test tubes with plugs, respectively, and the final volume of each tube was 2 mL with the deionized water. Afterward, tubes were added with 1 mL of phenol (5%) and 5 mL of concentrated H_2_SO_4_. The samples were gently mixed and kept for 15 mins at room temperature (RT). The absorbance was recorded at 485 nm using a spectrophotometer (Shanghai Mapada Co., Ltd., China). In general, the glucose standard curve was drawn with the standard glucose concentration (mg/mL) as the abscissa and the absorbance value as the ordinate. Finally, 5 mg of EFPs was thoroughly dissolved in 100 mL of deionized water and subjected to the same reaction as with the glucose standard solution. The absorbance was recorded at 485 nm and substituted into a glucose standard curve for estimating the polysaccharide concentration.

### 2.4. Monosaccharide Composition Assays

A combination of high-performance liquid chromatography (HPLC) and 1-phenyl-3-methyl-5-pyrazolone (PMP) was used to detect the monosaccharide compositions of EFPs as described previously [14]. In detail, 20 mg of polysaccharide was infiltrated using 2.0 mL of trifluoroacetic acid (2.0 mol/L) in a nitrogen-sealed tube at 110°C for two hours. In the next step, following cooling the reaction mixture to RT, centrifugation was performed at 1000 rpm for 5 mins. Afterward, the supernatant was transferred to the evaporation pan, and the trifluoroacetic acid was removed with 1.5 mL of methyl alcohol. After drying at atmospheric pressure, the residue was resuspended in 2 mL of deionized water and kept at 4°C till used.

Herein, 100 *μ*L of the sample solution, including hydrolyzed polysaccharide or monosaccharide, was added with 100 *μ*L each of aq. NaOH (0.3 mol/L) and PMP (0.5 mol/L) solutions. In the following step, the solutions were mixed, sealed, reacted at 70°C for 30 mins, and then cooled to ambient temperature. Next, a neutralization operation was performed via the addition of 100 *μ*L of HCl (0.3 mol/L) solution. Finally, the neutralization solution was extracted by using 3 mL of chloroform and the aqueous phase was leached with a 0.45 *μ*m membrane filter to obtain the filtrate, which was subjected to HPLC analysis. In individual test tubes, 10 different standard monosaccharide solutions were added at concentrations of 0.1, 0.5, 1, 2, 5, and 10 *μ*mol/L to obtain the calibration curve.

For conducting the HPLC analysis, separation was carried out on an InertSustain C18 column (4.6 mm × 250 mm, 5 microns), run at 42°C, using a flow rate (1.0 mL/min). The mobile phase contained potassium phosphate buffer (A, 0.05 mol/L, pH 6.9) and acetonitrile (B), and the elution concentration was 92% (A)-8% (B). UV peak detection was carried out at 254 nm. All the chromatography solvents/solutions were filtered through membrane filters (0.45 *μ*m, Millipore) and sonicated for degassing in an ultrasonic bath before use.

### 2.5. Antioxidant Activity Assays

#### 2.5.1. Scavenging Rate of DPPH Radical

Emanuel's method was applied to determine the scavenging rate of DPPH radical [15]. Herein, 2 mL of the polysaccharide solution at varying concentrations (0.1, 0.2, 0.4, 0.6, 0.8, and 1.0 mg/mL) was absorbed in the test tubes and added with 2 mL of the anhydrous ethanol and 2 mL of the DPPH ethanol (0.1 mmol/L) solution. After homogeneous mixing, the solution was placed in the dark for 30 mins at RT, and then, the absorbance was recorded at a wavelength of 517 nm. The experiment was carried out in parallel three times, where butylated hydroxytoluene (BHT), ASPs, and LBPs were chosen as the positive (+ve) controls. The rate of DPPH radical scavenging was evaluated by applying the following formula:

(1) $$\begin{array}{c} \text{DPPH scavenging rate }\left(\%\right)=\left[1-\;\frac{\left(\text{Ai}-\text{Aj}\right)}{A0}\right]\times 100, \end{array}$$

where *A*_*i*_ = absorbance of the mixture, *A*_*j*_ = absorbance of the sample solution, and *A*_0_ = absorbance of the DPPH solution.

#### 2.5.2. Inhibition Capacity of Hydroxyl Radical

The hydroxyl radical assay kit was applied to evaluate the inhibition potential of hydroxyl radical as per the Fenton reaction [16]. For this, individual test tubes were added with the sample solution at varying concentrations (0.05, 0.1, 0.15, 0.2, 0.3, and 0.4 mg/mL) and subsequently mixed with the different working solutions from the kit as per the provided instructions. The solutions were placed at RT for 20 mins, and then, their absorbance was recorded at a wavelength of 550 nm using the deionized water as the blank. The experiment was carried out in parallel three times, with vitamin C (VC), ASPs, and LBPs serving as +ve controls. The rate of hydroxyl radical inhibition was evaluated by applying the following formula:

(2) $$\begin{array}{c} \text{Hydroxyl radical inhibition rate}\left(\%\right)=\frac{\left({\text{OD}}_{\text{control}}\;-\;{\text{OD}}_{\text{determined}}\right)}{{\text{OD}}_{\text{control}}}\times 100. \end{array}$$

#### 2.5.3. Inhibition Capacity of Superoxide Anion Radical

The superoxide anion radical assay kit was applied to test the inhibition capacity of the superoxide anion radical [17]. For this, individual test tubes were added with the sample solution at varying concentrations (0.05, 0.1, 0.15, 0.2, 0.3, and 0.4 mg/mL) and subsequently mixed with different working solutions from the kit as per instructions. The solutions were placed at RT for 20 mins, and then, the absorbance was recorded at a wavelength of 550 nm using the deionized water as the blank. The experiment was carried out in parallel three times, with VC, ASPs, and LBPs serving as +ve controls. The rate of superoxide anion radical inhibition was evaluated by applying the following formula:

(3) $$\begin{array}{c} \text{Superoxide anion radical inhibition rate}\left(\%\right)=\frac{\left({\text{OD}}_{\text{control}}\;-\;{\text{OD}}_{\text{determined}}\right)}{{\text{OD}}_{\text{control}}}\times 100. \end{array}$$

#### 2.5.4. Determination of Reducing Capacity

The reducing capacity of EFPs was determined by implementing Prussian Blue's method [18]. For this, the individual test tubes were added with the sample solution at varying concentrations (0.05, 0.1, 0.15, 0.2, 0.3, and 0.4 mg/mL) and subsequently added with 2.5 mL each of the phosphate buffer (0.2 mol/L) and 1% of the potassium ferricyanide solution. After homogeneous mixing, the solutions were kept for 20 mins in a 50°C water bath. Afterward, the solutions were brought to RT and immediately added with 2.5 mL of trichloroacetic acid (10%). The blending solutions were subjected to centrifugation at 3,000 rpm for 10 mins, and the individual supernatants were separated. Finally, the absorbance of the mixture, including the supernatant, deionized water, and 0.1% of the trichlorinated iron solution, was measured at 700 nm by setting the deionized water as the blank. The experiment was measured in parallel three times, and BHT, ASPs, and LBPs were chosen as +ve controls.

### 2.6. Cell Proliferation and Cycle Assays

The proliferation of EFP-treated SKOV3 cells was assessed by means of the CCK-8 method as described earlier [19]. For this, the SKOV3 cell resuspension was carried out in the RPMI 1640 medium containing 10% of the fetal bovine serum. Then, the cells were diluted to 1.0 × 10^5^ cells/mL using the serum-free medium and inoculated into 96-well tissue culture plates. Following incubation with the EFPs at different concentrations (200, 400, and 800 *μ*g/mL) at 37°C for 24 h, 48 h, 72 h, and 96 h, respectively, in a 5% CO_2_ humidified atmosphere, cell proliferation was assessed by measuring absorbance at 450 nm using a microplate reader (Bio-Rad, USA). Each experiment was carried out independently at least three times. The inhibition rate of cell proliferation on polysaccharide treatment was calculated according to the following formula:

(4) $$\begin{array}{c} \text{The inhibition rate}\left(\%\right)=\left[\frac{\left(B-A\right)}{\left(B-C\right)}\right]\times 100, \end{array}$$

where *A* is the absorbance of the experimental group, *B* is the absorbance of the control group, and *C* is the absorbance of the blank group.

A cell cycle distribution for EFP-treated SKOV3 cells was examined using the Annexin V-FITC/PI staining method. Briefly, the cells were plated using a density of 5.0 × 10^5^ cells/well and subjected to EFP treatment procedures at concentrations (200, 600, and 1000 *μ*g/mL) for 72 h. Following centrifugation, the cell precipitates were collected and resuspended in the precooled absolute ethanol added slowly. After being fixed away from light at 4°C for more than 2.5 h, the cell precipitates were collected by centrifugation and resuspended in the precooled phosphate buffer saline. This cell resuspension solution was again centrifuged to collect the cell precipitates, to which PI staining solution (PI 50 mg/L and RNase A 1 g/L) was added and placed in the dark at RT for 15 mins. Lastly, the cell cycle was evaluated using a Guava easyCyte flow cytometry with a Guavasoft 3.1.1 (BD, USA).

### 2.7. Statistical Analysis

All statistical analyses were carried out using SPSS (IBM SPSS Statistics for Windows, version v23.0 (IBM Corp., Armonk, NY, USA)) and plotted with GraphPad Prism (v7.0) software. When following normal distributions, the data were analyzed using Student's *t*-test, or else, the nonparametric Mann–Whitney test was applied. A *p* < 0.05 (or less) was taken to indicate a statistically significant difference. Measurement data were expressed as the mean ± standard deviation (SD).

## 3. Results

### 3.1. SFE for EFP Extraction Parameters

**Figure 3:**
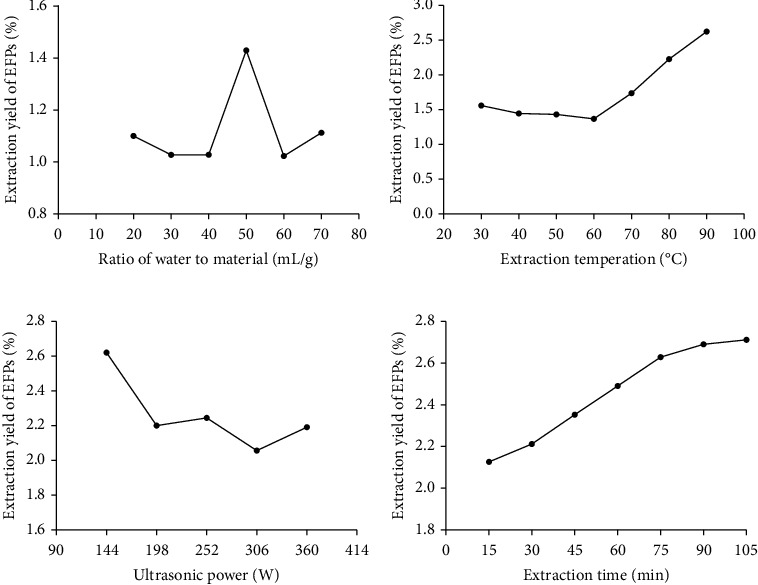
The effects of different factors on the ER of EFPs. (a) The effects of different material-liquid ratios, (b) the effects of different extraction temperatures, (c) the effects of different ultrasonic power, and (d) the effects of different extraction times.

The effects of various extraction parameters on the ER of EFPs are illustrated in Figures 3(a)–3(d). As shown in Figure 3(a), when the material-liquid ratio was 50 mL/g, the polysaccharide extraction rate (PER) reached a maximum, but this phenomenon was reversed on the material-liquid ratio exceeding 50 mL/g, indicating 50 mL/g as an optimum level for the next experiments. In Figure 3(b), when the temperature was 60°C, the PER increased obviously and it increased with the increase in temperature. According to the heat that could destroy the structure of polysaccharides, 70°C has been selected as an alternative temperature for EFP extraction. Furthermore, Figure 3(c) depicts a decrease in PER on increasing ultrasonic power. With a view to the influence of other components in EFPs and the excessive ultrasonic power that could destroy the structure of polysaccharides, 144 W was selected as an alternative ultrasonic power for EFP extraction.  Figure 3(d) illustrates that with an increase in extraction time from 15 to 105 mins, the PER increased, but the onward PER slowed down with the increase in extraction time from 75 to 105 mins. Thus, considering time and benefit, 75 mins was chosen for future experiments.

### 3.2. Orthogonal Test Analysis

The outcomes from extreme differences reveal the influence of four factors on the EFP yield in order as follows: water to extraction temperature (*R* = 1.607) > ultrasonic power (*R* = 0.590) > extraction time (*R* = 0.484) > material-liquid ratio (*R* = 0.210) (Table 2). The results were in line with F values from the analysis of variance (ANOVA) (Table 3). According to ANOVA, the influence of four factors (*p* < 0.01) on the EFP yield was highly significant. Based on extreme differences and ANOVA, the optimum parameters influencing EFP extraction involved the water-to-material-liquid ratio of 60 mL/g, the extraction temperature of 80°C, the ultrasonic power of 144W, and the extraction time of 75 mins. A verification test was performed three times under these conditions to determine the highest yield, and this EFP yield reached 2.63%. After deproteinization, the polysaccharide content was 53.47%. The powdered polysaccharides were then used for further monosaccharide compositions and antioxidant activity testing, thus evaluating the effect on the proliferation and the cycle of SKOV3 cells.

### 3.3. Monosaccharide Composition

**Figure 4:**
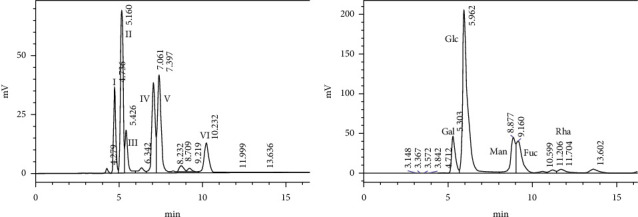
(a) Standard monosaccharide mixture. I: Gal; II: Glc; III: Ara; IV: Man; V: Fuc; VI: Rha. (b) The monosaccharide compositions of EFPs.

The existence of monosaccharides was validated by comparing the outcomes of the PMP derivatives of standard monosaccharides (Figure 4(a)). As shown in Figure 4(b) and Table 4, the EFPs were composed of Gal, Glc, Man, Fuc, and Rha. The monosaccharide contents and the molar ratios of EFPs are listed in Table 4, which shows the highest Glc and lowest Rha contents.

### 3.4. The DPPH Radical Scavenging Capacity

**Figure 5:**
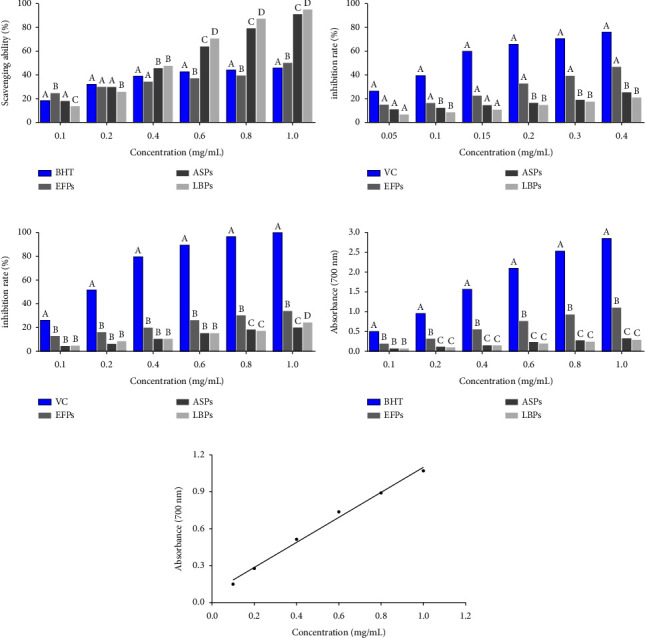
Scavenging activities on (a) DPPH, (b) hydroxyl radical, (c) superoxide anion radical, and (d and e) reducing power assay for EFPs at various concentrations. Different uppercase letters for the same concentration indicated a significant difference, *p* < 0.05; the same uppercase letters at the same concentration indicated no significant difference, *p* > 0.05. Data shown are the mean ± standard deviation (*n* = 3).

Figure 5(a) illustrates the DPPH scavenging activity of EFPs at varying concentrations. The results showed that EFPs scavenged DPPH in a concentration-dependent manner, whereas the inhibition rate increased from 24.00 to 49.00% in the range of 0.1 mg/mL to 1.0 mg/mL. The DPPH scavenging activity of EFPs was weaker than that of ASPs and LBPs at the concentration of 0.4 mg/mL to 1.0 mg/mL (*p* < 0.05), but it was similar to that of BHT, a commonly used antioxidant, which was consistent with the result of the IC_50_ values shown in Table 5.

### 3.5. The ·OH Radical Scavenging Capacity

The ·OH scavenging activity of EFPs at various concentrations is illustrated in Figure 5(b). The EFPs demonstrated scavenging activity on ·OH in a concentration-dependent manner, whereas the inhibition rate was found to increase from 11.30 to 64.20% at a concentration range of 0.05–0.4 mg/mL. The ·OH scavenging activity of EFPs was weaker than that of VC, which is a commonly used antioxidant, but it was higher than that of ASPs and LBPs at the concentration range of 0.2 mg/mL to 0.4 mg/mL (*p* < 0.05), which was also consistent with the IC_50_ values shown in Table 5.

### 3.6. The Superoxide Anion Radical Scavenging Capacity

As illustrated in Figure 5(c), the EFPs demonstrated superoxide anion scavenging activity in a concentration-dependent manner, whereas the inhibition rate was found to increase from 11.60% to 32.89% at a concentration range of 0.1–1.0 mg/mL. Also, the ability of EFPs to scavenge superoxide anion was lower than that of VC but higher than that of ASPs and LBPs at the concentration range of 0.8–1.0 mg/mL (*p* < 0.05), which was consistent with the result of the IC_50_ values shown in Table 5.

### 3.7. Reducing Power Assay

As shown in Figure 5(e), linear growth of reducing power was observed with increasing sample concentration at a concentration range of 0.1 to 1.0 mg/mL (*r* = 0.996 5). The dose-dependent effect of EFPs on reducing power is illustrated in Figure 5(d). At 0.2 mg/mL to 1.0 mg/mL, EFPs exhibited a lower reducing power than BHT, but a higher power than ASPs and LBPs at the same concentration (*p* < 0.05).

### 3.8. *In Vitro* Analysis of Anti-Ovarian Adenocarcinoma Activity for EFPs via CCK-8 Assay

**Figure 6:**
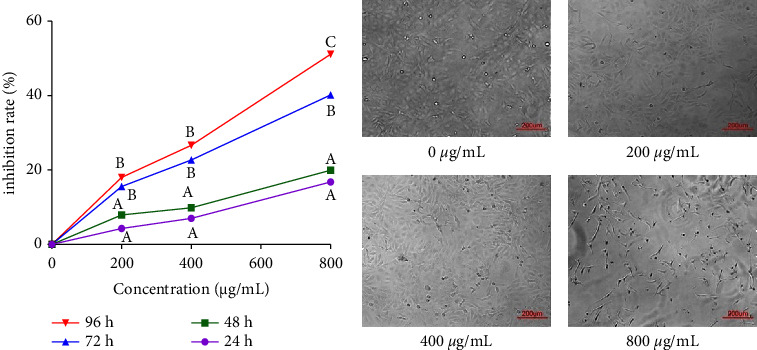
(a) The effect of EFPs at different concentrations and treatment times on the proliferation inhibition rate of SKOV3 cells. Different uppercase letters for the same concentration indicated significant differences, *p* < 0.05. (b) The morphological characteristics of the SKOV3 cells treated with different concentrations of EFPs for 72 h.

To identify the anti-ovarian adenocarcinoma activities of EFPs *in vitro*, the viability of SKOV3 cells on treatment with various concentrations of EFPs for varied periods (24 h, 48 h, 72 h, and 96 h) was assessed using the CCK-8 method.  Figure 6(a) illustrates an increase in the growth inhibition of SKOV3 cells with increasing EFP concentration and time. Compared with other time intervals, the EFPs have a considerable inhibitory effect on the inhibition rate of SKOV3 cell growth in the dose range of 200–800 *μ*g/mL at 48 and 72 hours, where the differences were statistically significant (*p* < 0.05). Therefore, EFPs in the concentration range (200, 400, and 800 *μ*g/mL) with a treatment duration of 72 h were selected for further anti-ovarian adenocarcinoma activity analyses. As shown in Figure 6(b), the SKOV3 cells possessing normal morphologies showed irregular spindle forms, large numbers, complete shapes, and clustered adherent growth. In comparison with the blank group (0 *μ*g/mL), the SKOV3 cells experienced a gradual loss of their original cellular morphology with an increase in EFP concentrations. This indicated the cell number declined considerably, becoming stripped with gradually increased floating.

### 3.9. EFPs Suppressed the Cell Cycle and Promoted Apoptosis of SKOV3 Cells

**Figure 7:**
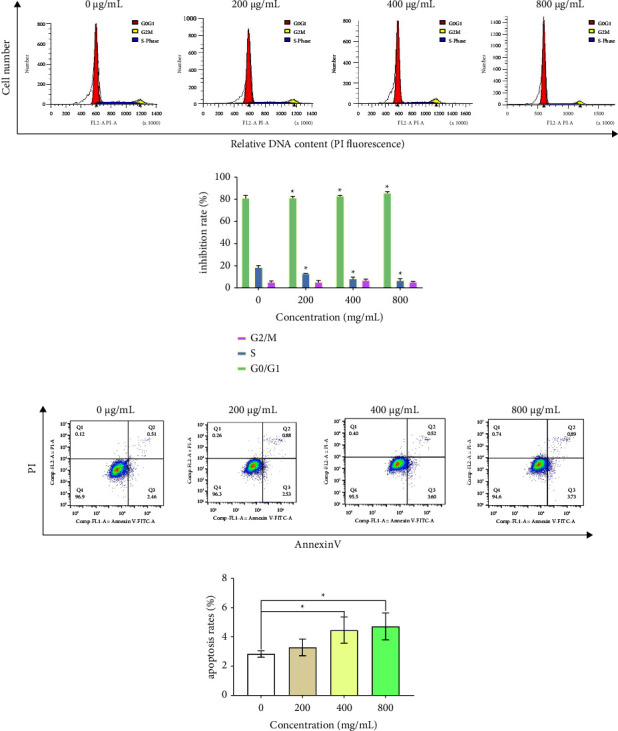
The DNA contents in the cell cycle were measured quantitatively by flow cytometry (a), and the number of apoptotic cells was determined by Annexin V-FITC/PI double staining (b). Compared to the 0 *μ*g/mL group, ^*∗*^*p*  <  0.05.

The EFP effect on the cell cycle of SKOV3 cells was examined by analyzing DNA amount (%) at the G0/G1, S, and G2/M phases of the cell cycle. In comparison with the blank group (0 *μ*g/mL), the proportion of cells in G1-phase cells increased from 81.49% ± 2.01 to 85.93% ± 1.01 (*p*  <  0.05) in the concentration range of EFPs (200, 400, and 800 *μ*g/mL) with a treatment duration of 72 h, whereas the value in S-phase cells declined from 18.82% ± 1.22 to 6.90 ± 1.50% (*p*  <  0.05) demonstrating the concentration-dependent effect, whereas the effect in G2/M phase cells was considerably insignificant (Figure 7(a) and Table 6). Following treatment with EFPs for 72 h, the total apoptosis rates of the blank group (0 *μ*g/mL) and the treatment group (400 *μ*g/mL and 800 *μ*g/mL) were 4.46% and 4.71%, respectively (Figure 7(b) and Table 7), with statistically significant differences (*p* < 0.05).

## 4. Discussion

In this research, multiple experiments were performed to understand the monosaccharide composition and antioxidant and anticancer effects of EFPs. As we know, polysaccharides are among the most versatile natural active substances due to their wide variety of pharmacological activities such as antiaging [20–22], liver injury protection [23–25], blood glucose lowering [26–29], antifatigue [30, 31], immunoregulatory activity [32–35], and antitumor activity [36–39]. Presently, frequently used extraction methods for polysaccharides mainly include hot water extraction [40], alcohol extraction [40], ultrasonic-assisted extraction [41], microwave-assisted extraction [42], and infrared-assisted extraction [43]. Compared with the other extraction methods, ultrasonic extraction was chosen to carry out a single-factor test as this method could break up the plant cell walls and accelerate the diffusion and dissolution of polysaccharides. Importantly, this method had the advantages of simple operation, low cost, and stable extraction. The optimum extraction conditions were obtained by SFE (material-liquid ratio: 50 mL/g, temperature: 70°C, ultrasonic power: 144 W, and extraction time: 75 mins). However, the single-factor experiments just considered the influence of each factor on the extraction yield and did not involve the interactions among them, as well as the influence of each factor on the extraction yield. Therefore, the optimal experimental range of material-liquid ratios, extraction temperature, ultrasonic power, and extraction time was determined by the orthogonal test [44, 45]. In the orthogonal test, only nine experiments were conducted for four factors at three levels each, whereas 81 experiments would be necessary for the full-factorial design. At last, A_2_B_2_C_1_ was reasonably confirmed to be the best optimum combination of different parameters. After deproteinization, the polysaccharide content was 53.47%, which was no less than that of mushroom polysaccharides [46], indicating that it may have a similar function to mushroom polysaccharides.

To analyze the monosaccharide compositions, we chose the HPLC method, which has the advantages of fast separation speed, high resolution, good precision and reproducibility, and high stability [47–49]. It was shown that EFPs have five main monosaccharide components, including Gal, Glc, Man, Fuc, and Rha. It has been extensively reported that ASPs and LBPs are commonly used natural antioxidants [50, 51]. ASPs are composed of five monosaccharides, which are Rha, Gal acid, Glc, Gal, and Ara, whereas LBPs are composed of four monosaccharides, viz., Rha, Man, Glc, and Gal. Moreover, Deng [52] and Zhang [53], respectively, found that both ASPs and LBPs have antioxidant and antitumor activities. Numerous research studies revealed that mushroom polysaccharides also have antioxidant and antitumor activities [46, 54, 55]. Therefore, it was speculated that EFPs could also have antioxidant and antitumor activities. In this study, no unreported monosaccharides have been found in EFPs, but we initiatively extracted EFPs for antiovarian cancer research, which could provide a new direction on more anticancer effects of *E. fortunei*.

To prove the antioxidant activity of polysaccharides, DPPH, ·OH, superoxide anion, and reducing power analysis were performed in the following experiments. DPPH as a stable free radical in organic solvents is commonly used to evaluate the ability of natural compounds to scavenge free radicals [56, 57]. Usually, the DPPH free radical inhibition rate is measured to evaluate their free radical scavenging ability and antioxidant capacity. In reactive oxygen species, hydroxyl radicals are among the most harmful radicals, causing severe oxidative damage to nearby biomolecules (such as carbohydrates, proteins, lipids, and DNA) [58, 59]. Superoxide radicals are highly toxic substances produced during various biological and photochemical reactions and also play an important role in the formation of other reactive oxygen species [58, 59]. Reducing power is a key indicator for evaluating the antioxidant activity of substances. Antioxidant substances give electrons by their reduction and scavenge free radicals to interrupt the chain reaction of lipid peroxidation. Antioxidant strength can be evaluated by measuring the reducing power [58, 59]. In this study, the DPPH scavenging activity of EFPs was weaker than that of ASPs and LBPs, but it was similar to that of BHT. In addition, the ·OH and superoxide anion scavenging activity of EFPs was weaker than that of VC, but it was higher than that of ASPs and LBPs. Also, EFPs exhibited a lower reducing power than BHT, but a higher power than ASPs and LBPs at the same concentration. In short, it suggested that EFPs and other polysaccharides had potential antioxidant activity to be developed as antioxidants *in vitro*.

It has been widely reported that plant polysaccharides possessing antioxidant properties may prevent tumor cells from proliferating. Based on the published literature, the antitumor activities of polysaccharides are associated with their antioxidant and free radical scavenging activities. ASPs could inhibit the proliferation of both lung cancer cells (A549) and liver cancer cells (HepG2) [60, 61]. LBPs could also result in the inhibition of MCF-7 and HepG2 cell proliferation, thus inducing apoptosis of both types of cells [62, 63]. Polysaccharides from *Pleurotus ostreatus* may reduce the viability of A549, SKOV3, HepG2, and MCF-7 cells in a concentration-dependent manner while inducing apoptosis of A549 cells [64]. *Duchesnea indica (Andr.) Focke* polysaccharides significantly inhibited the proliferation of SKOV3 and HepG2 cancer cell lines [65]. Polysaccharides from *Cordyceps gunnii* could significantly induce apoptosis in SKOV3 cells through the p53-Bax-caspase pathway [66]. In the above research, we found that EFPs have antioxidant effects. Therefore, we speculated that the antioxidant EFPs also have certain antitumor effects and took it as the next research direction. In the present research, the changes in cell viability and morphology illustrated an increase in the growth inhibition of SKOV3 cells with increasing EFP concentration and time. Moreover, the SKOV3 cells were arrested by EFPs at the G0/G1 and S phases of the cell cycle and the total apoptosis rate increased in the treatment group (400 *μ*g/mL and 800 *μ*g/mL) with statistically significant differences (*p* < 0.05). These outcomes illustrated that EFPs could induce apoptosis and inhibit the proliferation of SKOV3 cells *in vitro*. We speculated that EFPs could block the division of SKOV3 cells in the G0/G1 phase, affect DNA replication and transformation into S and M phases, and ultimately slow down the growth of SKOV3 cells to reduce their proliferative activity and lead to cell apoptosis.

Notably, Sudha Govindan [54] found that *Hypsizygus ulmarius* polysaccharides exhibited antioxidant, liver protective, and lipid-lowering activities in ethanol-induced rats. Nataraj et al. [46] found that the antioxidant properties of crude polysaccharides from *Calocybe indica* (CICP) were assessed using a variety of in vitro tests and also inhibited the growth of HeLa, PC3, HT29, HepG2, and Jurkat cells in a concentration-dependent manner. The results of Shen et al. [63] revealed that LBPs caused growth inhibition of MCF-7 cells with the arrest of the cell cycle in the S phase and induced apoptosis through the ERK pathway. The results of Li et al. [35] revealed that anticancer activity in vivo of ASPs may be achieved by restoring immune organs, regulating cellular immune responses, and increasing cytokine levels. Compared with the above traditional Chinese medicine or medicinal plant polysaccharides, EFPs have similar polysaccharide composition and in vitro antioxidant and antitumor effects, indicating that they could be used as the potential natural antioxidant and antitumor agents. However, the molecular mechanism needs further study. Of course, we need to find out the genes or proteins related to the effect of EFPs on the proliferation and apoptosis of ovarian cancer cells, discover their signaling pathway, and reveal the specific mechanism.

## 5. Conclusions

This study, for the first time, used a single factor combined with an orthogonal experiment to detect the factors influencing EFP extraction to obtain optimal extraction conditions. The results showed that the optimal parameter combination of EFPs was material-liquid ratios of 60 mL/g, extraction temperature of 80°C, ultrasonic power of 144 W, and extraction time of 75 mins. In this condition, the highest yield of 2.63 ± 0.23% was obtained. After deproteinization 3 times, the polysaccharide content increased and the protein content decreased, respectively. Subsequently, we comprehended the monosaccharide composition of the polysaccharides, including Gal, Glc, Man, Fuc, and Rha. Furthermore, the antioxidant capacity of EFPs *in vitro* was analyzed using five different evaluation methods and the results showed that EFPs have a certain antioxidant capacity, which was also superior to that of ASPs and LBPs to a certain extent. Finally, the effects of various polysaccharide concentrations on the proliferation of SKOV3 cells, cell cycle, and apoptosis were evaluated. The cell proliferation was inhibited following treatment with different concentrations of EFPs (200, 400, and 800 *μ*g/mL). Also, there was a cell arrest by EFPs at the G0/G1 and S phases of the cell cycle, which illustrates that EFPs can induce apoptosis. These results demonstrate that polysaccharides isolated and purified from *E. fortunei* exert *in vitro* antitumor activity, which deserves further examination to investigate the antitumor mechanisms of EFPs. However, we did not detect the expression of apoptosis-related genes and proteins, reveal the signaling pathway, and clarify the anticancer mechanism. Therefore, further works are focused on the expressions of caspase-3, caspase-9, PARP, *p*53, Bax, and Bcl-2 in the SKOV3 cells, which were studied via Western blotting, and animal experiments revealed whether EFPs inhibit tumors within an ovarian tumor model rat, modeled with the SKOV3 cells.

## Figures and Tables

**Table 1:** Factors and levels used in the orthogonal experimental design.

Variable	Level		
	1	2	3
(A) Material-liquid ratios (mL/g)	40	50	60
(B) Extraction temperature (°C)	60	70	80
(C) Ultrasonic power (W)	144	198	252
(D) Extraction time (min)	60	75	90

**Table 2:** Orthogonal test design and results for the ER of EFPs (*n* = 3).

No.	Factors				ER (%)
	A	B	C	D	
1	1	1	1	1	1.860 ± 0.001
2	2	1	2	2	1.840 ± 0.017
3	3	1	3	3	1.810 ± 0.023
4	2	2	1	3	2.117 ± 0.017
5	3	2	2	1	1.927 ± 0.043
6	1	2	3	2	2.047 ± 0.021
7	3	3	1	2	2.627 ± 0.041
8	1	3	2	3	2.247 ± 0.021
9	2	3	3	1	2.243 ± 0.021
*k*1	6.154	5.510	6.604	6.030	
*k*2	6.200	6.091	6.014	6.514	
*k*3	6.364	7.117	6.100	6.174	
*R*	0.210	1.607	0.590	0.484	

A: material-liquid ratio; B: extraction temperature; C: ultrasonic power; D: extraction time.

**Table 3:** Variance analysis of the orthogonal test.

Factors	SS	*df*	MS	*F* value	*P* value	Significant
A	0.024	2	0.012	18.293	≤0.001	^*∗∗∗*^
B	1.324	2	0.662	994.000	≤0.001	^*∗∗∗*^
C	0.203	2	0.102	152.505	≤0.001	^*∗∗∗*^
D	0.124	2	0.062	92.725	≤0.001	^*∗∗∗*^
Deviation	0.012	18	0.001			

SS: sum of square; *df*: degree of freedom; MS: mean of square; ^*∗∗∗*^: *p* ≤ 0.001; A: material-liquid ratio; B: extraction temperature; C: ultrasonic power; D: extraction time.

**Table 4:** Monosaccharide composition of EFPs.

Number	Monosaccharide	Contents (mg/g)	Molar ratio
1	Gal	12.675	7.14
2	Glc	42.560	23.99
3	Man	11.162	6.29
4	Fuc	11.622	6.55
5	Rha	1.774	1.00

Gal: galactose; Glc: glucose; Man: mannose; Fuc: fucose; Rha: rhamnose.

**Table 5:** The IC_50_ values of various polysaccharide samples from antioxidant properties.

Sample	IC_50_ value (mg/mL)		
	DPPH scavenging activity	·OH scavenging activity	O_2_^·−1^ scavenging activity
EFPs	1.751 ± 0.03^b^	0.481 ± 0.01^b^	3.441 ± 0.22^a^
ASPs	0.379 ± 0.02^a^	3.708 ± 0.15^a^	4.985 ± 0.50^a^
LBPs	0.373 ± 0.01^a^	2.680 ± 0.14^a^	4.305 ± 0.29^a^
BHT	1.231 ± 0.03		
VC		0.381 ± 0.49	0.219 ± 0.12

The IC_50_ value is half the maximal inhibitory concentration. Data are presented as mean ± standard deviation (*n* = 3). The superscript letters a and b indicate a significant difference at the 0.05 significance level.

**Table 6:** The EFP effect on the cell cycle distribution of SKOV3 cells treated for 72 h (*n* = 3).

Cell cycle (%)	Concentration (*μ*g/mL)			
	0	200	400	800
G0/G1	81.49 ± 2.01	81.53 ± 1.17^*∗*^	83.17 ± 0.36^*∗*^	85.93 ± 1.01^*∗*^
S	18.82 ± 1.22	13.01 ± 0.12^*∗*^	8.57 ± 1.18^*∗*^	6.90 ± 1.50^*∗*^
G2/M	5.36 ± 0.99	5.46 ± 1.29	7.06 ± 0.95	5.50 ± 0.34

*Note.* The SKOV3 cells were treated with EFPs (200 *μ*g/mL, 400 *μ*g/mL, and 800 *μ*g/mL) for 72 h (mean ± SD, ^*∗*^*p*  <  0.05 vs. non-EFP-treated group).

**Table 7:** The EFP effect on the cell apoptosis of SKOV3 cells treated for 72 h (*n* = 3).

Concentration (*μ*g/mL)	Apoptosis (%)
0	2.83 ± 0.22
200	3.28 ± 0.57
400	4.46 ± 0.90^*∗*^
800	4.71 ± 0.92^*∗*^

*Note.* SKOV3 cells were treated with EFPs (200 *μ*g/mL, 400 *μ*g/mL, and 800 *μ*g/mL) for 72 h (mean ± SD, ^*∗*^*p*  <  0.05 vs. non-EFP-treated group).

## Data Availability

The original contributions presented in the study are included in the article, and further inquiries can be directed to the corresponding author.
